# SARS-CoV-2 Omicron Variant: Epidemiological Features, Biological Characteristics, and Clinical Significance

**DOI:** 10.3389/fimmu.2022.877101

**Published:** 2022-04-29

**Authors:** Yifei Guo, Jiajia Han, Yao Zhang, Jingjing He, Weien Yu, Xueyun Zhang, Jingwen Wu, Shenyan Zhang, Yide Kong, Yue Guo, Yanxue Lin, Jiming Zhang

**Affiliations:** ^1^ Department of Infectious Diseases, Shanghai Key Laboratory of Infectious Diseases and Biosafety Emergency Response, Shanghai Institute of Infectious Diseases and Biosecurity, National Medical Center for Infectious Diseases, Huashan Hospital, Fudan University, Shanghai, China; ^2^ Key Laboratory of Medical Molecular Virology (MOE/MOH), Shanghai Medical College, Fudan University, Shanghai, China; ^3^ Department of Infectious Diseases, Jing’An Branch of Huashan Hospital, Fudan University, Shanghai, China

**Keywords:** COVID-19, SARS-CoV-2 variants, mutations, neutralizing antibodies, vaccines, sublineages

## Abstract

The SARS-CoV-2 Omicron (B.1.1529) variant was designated as a variant of concern (VOC) by the World Health Organization (WHO) on November 26, 2021. Within two months, it had replaced the Delta variant and had become the dominant circulating variant around the world. The Omicron variant possesses an unprecedented number of mutations, especially in the spike protein, which may be influencing its biological and clinical aspects. Preliminary studies have suggested that increased transmissibility and the reduced protective effects of neutralizing antibodies have contributed to the rapid spread of this variant, posing a significant challenge to control the coronavirus disease 2019 (COVID-19) pandemic. There is, however, a silver lining for this wave of the Omicron variant. A lower risk of hospitalization and mortality has been observed in prevailing countries. Booster vaccination also has ameliorated a significant reduction in neutralization. Antiviral drugs are minimally influenced. Moreover, the functions of Fc-mediated and T-cell immunity have been retained to a great extent, both of which play a key role in preventing severe disease.

## Introduction

Over the past two years, severe acute respiratory syndrome coronavirus 2 (SARS-CoV-2) has spread rapidly around the world. It has caused more than 479 million confirmed cases of coronavirus disease 2019 (COVID-19) cases and 6 million deaths worldwide (1). The SARS-CoV-2 virus has evolved continuously since its emergence. During late 2020, a variant named B.1.1.7 emerged and spread rapidly (2). Subsequently, to alert the public, the World Health Organization (WHO) categorized these variants into variants of concern (VOCs) and variants of interest (VOIs).

**Figure 1:**
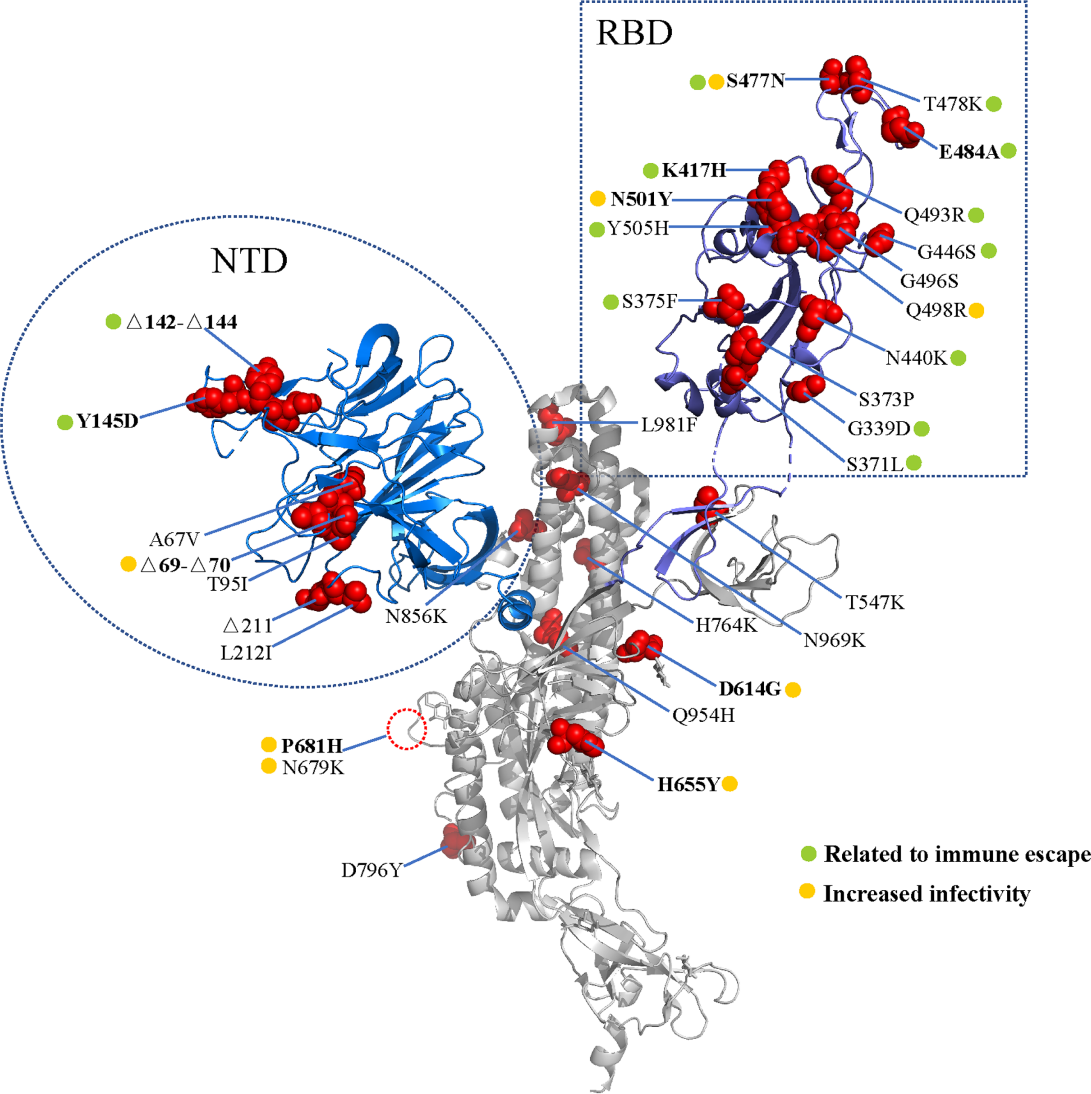
Spike mutations in the Omicron variant. Cartoon representation of the severe acute respiratory syndrome coronavirus 2 (SARS-CoV-2) spike protein (PDB: 7CWU_A). Red spheres represent the mutations found in the Omicron variant. The receptor binding domain (RBD) and amino terminal domain (NTD) are shown in blue and purple, respectively. The mutations are marked according to the immune escape (green) and increased infectivity (yellow).

In November 2021, the B.1.1.529 variant was first reported in South Africa and Botswana. This variant was found to possess at least 35 mutations in the spike protein, and increasing cases in South Africa were consistent with the detection of the B.1.1.529 variant (3, 4) (**Figure 1**). The WHO designated the B.1.1.529 variant as a new VOC, named the Omicron variant. It is predicted that the Omicron variant will infect more than 50% of the world population (5).

In this review, we will summarize the epidemiological features, biological characteristics, and clinical significance of the Omicron variant.

## 1 Omicron Variant: Burst Onto the Scene and Spread Worldwide Rapidly

The emergence of the Omicron variant is seemingly an unexpected event, because it likely did not evolve from other known circulating SARS-CoV-2 variants (4). Notably, the Omicron variant was first detected in Africa, but the exact origin has not been confirmed. Through time-calibrated Bayesian phylogenetic analysis, the most recent common progenitors may have been present in early October 2021 (6). As for the confusing origin of the Omicron variant, there are three main explanations. First, the Omicron variant may spread and evolve in the blind area of surveillance systems. Second, the Omicron variant may evolve in immunocompromised patients. This latter assumption is based on clinical observations. For example, the ancestral virus existed for more than six months in an advanced acquired immune deficiency syndrome (AIDS) patient and some mutations evolved that were associated with an immune escape effect (7). Third, the Omicron variant may have come from the virus reservoirs of rodents or other hosts. Some mutations, such as Q493R and Q498R, which are uncommon in circulating variants, also were found in mouse-adapted variants (8).

As of 31 March, 2022, the Omicron variant had been detected in 188 countries and already had become the dominant strain on a global scale, accounting for 99.7% submitted sequences from 23 February to 24 March 2022 (1, 9). The Omicron variant has diverged into four sublineages: BA.1, BA.1.1, BA.2, and BA.3 (6, 10). Most of the circulating Omicron variants are BA.1, BA.1.1 and BA.2. The Omicron BA.1 variant, also known as the original form, can be identified by S-gene target failure (SGTF). The Omicron BA.1.1 variant is a subvariant of BA.1 with an R346K mutation. Notably, the proportion of BA.2, which cannot cause SGTF, has been on the rise and the Omicron BA.2 variant has become dominant in many countries such as Denmark, India, Norway, Singapore, indicating it may have a selective advantage over the Omicron BA.1 variant (11). One epidemiological study in Denmark suggested that the effective reproduction number of BA.2 was about 1.26 times larger than that of BA.1 (12) (**Table 1**).

**Table 1:** The differences and similarities between the Omicron BA.1 and BA.2 variants.

		BA.1	BA.2	Ref.
**Difference**				
Unique spike mutations	RBD	S371L, G446S, G496S, R346K(BA.1.1)	S371F, T376A, D405N, R408S	(2)
	NTD	A67V, Del69-70, T95I, Del143-145, Del211, L212I, Ins214EPE	T19I, Del24-26, A27S, V213G	
	Other domains	T547K, N856K, L981F	——	
S-gene target failure^a^		Yes	No	(13)
Generation time^b^		2.77 - 2.91 days	2.35 - 2.44 days	(12, 14)
Effective reproduction number (Re)^c^ R_Omicron/_R_Delta_		1.99 (95%CI: 1.98 - 2.02) times	2.51 (95%CI: 2.48 - 2.55) times	(12)
Effective therapeutic monoclonal antibody		S309 (sotrovimab)	COV2-2130 (cilgavimab)	(15–18)
**Similarity**				
Common spike mutations	RBD	G339D, S373P, S375F, K417N, N440K, S477N, T478K, E484A, Q493R, Q498R, N501Y, Y505H		(2)
	NTD	G142D		
	Other domains	D614G, H655Y, N679K, P681H, N764K, D796Y, Q954H, N969K		
Clinical severity		Similar risks of hospitalization and severe diseases		(19)
Polyclonal antibody (vaccine/infection induced)		Similar neutralization titers against BA.1 and BA.2		(15, 20, 21)
Antiviral drugs		Still effective against BA.1 and BA.2		(16)

RBD, receptor-binding domain; NTD, amino terminal (N-terminal) domain; 95%CI, 95% confidence interval.

a S-gene is not detected by the real-time reverse transcriptase polymerase chain reaction (RT-PCR) testing methods. The Omicron BA.1 sublineage can be identified by RT-PCR testing because of Del69-70 in the spike protein.

b The interval between individuals becoming infected and transmitting the Omicron variant.

c Estimate the average number of infections generated by a case infected with the Omicron variant in a population that includes not only naïve people.

## 2 Omicron Variant Mutations

### 2.1 Omicron Variant: Key Amino Acid Mutations in the Spike Protein

SARS-CoV-2, including the Omicron variant, infects cells that rely on its obligate receptor-angiotensin-converting enzyme 2 (ACE2) (22–24). The entry process of the SARS-CoV-2 is mediated by furin cleaving the spike protein into two noncovalently associated subunits: the S1 subunit binds the ACE2, and the S2 subunit anchors the spike protein to the membrane and mediates subsequent membrane fusion (25). The S1 subunit consists of the receptor-binding domain (RBD), amino-terminal (N-terminal) domain (NTD), and two carboxy-terminal (C-terminal) domains (26). To better understand the biological characteristics of the Omicron variant, it is crucial to learn its amino acid mutations.

#### 2.1.1 RBD Mutations

**Figure 2:**
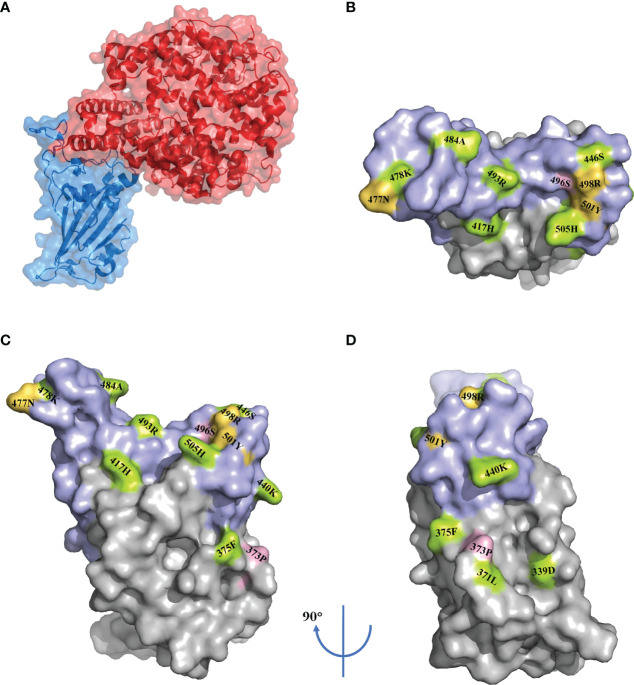
RBD mutations in the Omicron variant. Surface representation of the receptor-binding domain (RBD) in complex with the angiotensin-converting enzyme 2 (ACE2) (PDB: 7A94) **(A)**. Surface representation of the RBD mutations (PDB: 7A94) in three orientations. The receptor binding motif (RBM) is shown in purple. The mutations are marked according to the immune escape (green), increased infectivity (yellow) and unknown (pink) **(B–D)**.

RBD, part of the S1 subunit, binds to the ACE2, which is the fundamental first step for entry into the membrane. In addition, in one study of serological analyses among 650 individuals infected with SARS-CoV-2, 90% of the neutralizing antibodies targeted the RBD (27). Despite its crucial role, the selective pressure leads to the emergence of mutations for maintaining or increasing viral fitness. Shockingly, the Omicron variant carries 15 mutations in the RBD, whereas the Delta variant carries only two mutations in this area (4) (**Figure 2**). Of the 15 mutations, only four sites—E484A, N501Y, K417N, and T478K—were previously present in other VOCs.

The E484 residual has been identified as the immunodominant site on the RBD through a deep-mutational scanning method (28). Both the Beta variant and the Gamma variant have a substitution at E484 to K. The E484K substitution confers extensive resistance to monoclonal antibodies (mAbs) and plasma from convalescent or vaccinated individuals (28–34). In an escape mutation study, the E484A substitution in the Omicron variant possessed a similar escaping effect (35). In contrast to its vital impact on the resistance to neutralization, this binding affinity reduces significantly because the side chain of A484 is too short to come into contact with ACE2 (36).

The N501Y substitution is also present in the Alpha variant, Beta variant, and Gamma variant. Several studies have shown that N501Y confers increased ACE2 binding affinity (37–42). In one functional study on eight spike substitutions of the Alpha variant, only the N501Y substitution exhibited consistent replication capacity in hamsters and primary human airway epithelial cells, which indicated that N501Y might be one of the decisive sites increasing transmissibility (43). Notably, one study reported that Q498R was epistatic to N501Y (44). Because the N501Y substitution has a marginal impact on the availability of the spike epitopes, individual substitution at N501Y is less likely to be associated with immune escape (29, 37, 45).

The K417N substitution is also present in the Beta variant. It has been shown that the mutation at the site K417 reduces neutralization by some mAbs (40, 46, 47). K417N causes the absence of polar interaction with D30 from ACE2 and decreases the binding affinity (36).

The T478K substitution is a unique mutation of the Delta variant and is predominantly found in Mexico and the United States (48). Previous studies have shown that substitution at 478 retains the susceptibility to monoclonal or polyclonal antibodies (28, 46, 47).

#### 2.1.2 NTD Mutations

VOCs harbor numerous deletions and mutations in the NTD, which are associated with prolonged infection and escape from NTD-directed antibodies (49–52). Indeed, most of the potent NTD antibodies target a single “supersite”, where N3 hairpin (residual 141–156), N5 loop (residual 246–260), and the lower relative glycan density enhance immunogenicity. The Omicron variant bears deletions at residual 142–144, which most likely reduces susceptibility to NTD-directed antibodies (35, 49). △H69/V70 appears to not render the variant less sensitive to immune sera but enhances infection rates (53). Remarkably, △H69/V70 can lead to SGTF in real-time fluorescence quantitative polymerase chain reaction (RT-qPCR) assays, which has been used to rapidly distinguish the Omicron variant from the Delta variant (19).

#### 2.1.3 Mutations Close to the Furin Cleavage Site

Cleavage of the S1/S2 boundary by furin is necessary to initiate membrane fusion, which has implications for pathogenesis (25). In the absence of the furin cleavage site, viral replication significantly attenuates *in vitro* and *in vivo* experiments (54, 55). The substitutions adjacent to S1/S2 boundary—that is, P681H, H655Y, and N679K—are predicted to affect viral entry and to increase transmissibility (56–58).

### 2.2 Molecular Basis of Immune Evasion and Binding Capacity to ACE2

Numerous mutation sites related to immune escape have been identified by a multitude of studies. These sites are shown in **Figure 2** (4, 6, 35, 40, 47, 59, 60).

To some extent, the binding capacity of the RBD to ACE2 (i.e., the ACE2 binding affinity) reflects the infection rate of coronaviruses (61). Of the 15 mutations in the RBD of the Omicron variant, nine are located at the ACE2 binding interface: Y505H, N501Y, Q498R, G496S, Q493R, E484A, S477A, G446S, and K417N (60). Regarding the effect of a single mutation on ACE2 affinity, some mutations—including N501Y, S477N, and Q498R—increase the affinity, whereas others decrease the affinity (36, 60). Nevertheless, explanations of the overall impact on ACE2 binding affinity have been controversial. One study found a 2.4-fold increased binding capacity in comparison with the wild-type (WT) virus (62). In contrast to the view that the Omicron variant has increasing affinity, another study investigated the binding affinity of VOCs and the prototype SARS-CoV-2 by analyzing the crystal structure and found that the Delta and Omicron variants unexpectedly exhibited similar affinity to the prototype (36). Another study measured the affinity constant and found that the binding capacity of the Omicron variant was weaker than the Delta variant (63). Notably, because these results may be influenced by many factors, including the host and variant itself, they cannot completely represent real-world situations *in vivo* and may have weaker or higher infection rates.

## 3 Omicron Variant Appears to Cause Less Severe Disease

Preliminary clinical studies have reported that the rapidly spreading Omicron variant was less dangerous than its predecessor, the Delta variant. In South Africa, Wolter et al. found that people with SGTF infections (as a proxy for Omicron) had an 80% lower chance of being admitted to the hospital compared with people with non-SGTF infections (19). Among hospitalized patients, admission to intensive care and death rates were 18.5% versus 29.9% (p < 0.001) and 2.7% versus 28.1% (p < 0.001) for the Omicron wave and the Delta wave, respectively (64). In accordance with the analysis by the U.K. Health Security Agency, people infected with the Omicron variant were estimated to be between 50% and 57% less likely to present to emergency care than if they had been infected with the Delta variant and 30% to 37% less likely to be admitted to the hospital (65). In comparison, the hospitalization rates of children under 1 year old have risen rapidly, accounting for 42.4% of admissions, whereas they accounted for 30.2% of admissions during the period when the Delta variant was dominant. Less severity has been monitored in children (66). Additionally, the hospitalization rates of the omicron BA.2 variant are similar to that of the Omicron BA.1variant (67) (**Table 1**). It is hard to determine, however, whether the Omicron variant is less pathogenic than earlier variants because of the preexisting acquired or natural immunity and its limited spread into the elderly population.

In addition to clinical observations, researchers have tried to identify Omicron’s pathogenicity using *in vitro* and *in vivo* models. In one cell culture study, the Omicron variant replicated poorly in the Calu-3 cell line with a high expression of transmembrane serine protease 2 (TMPRSS2), whereas the Delta variant replicated well in this cell line (68). TMPRSS2-mediated spike protein activation induces ACE2-mediated endocytosis and then initiates fusion pore formation (25). TMPRSS2 and ACE2 were co-expressed in type II pneumocytes, and the Delta variant grew more rapidly and replicated well inside people’s lungs and throats (69, 70). These results suggested that the Omicron variant may have poorer replication in the lungs and be less risky than the Delta variant. Likewise, primary 3D lower airway organoids were applied to evaluate the entry efficiency of SARS-CoV-2, and the Omicron variant exhibited weaker infection rates relative to the Delta variant and the Wuhan/D614G strain. Thus, reduced access to the lower respiratory tract might mean milder symptoms than experienced with other circulating VOCs (71). The hamster is a suitable experimental animal model for exploring SARS-CoV-2 infections, as the pathogenic patterns of the SARS-CoV-2 virus in hamsters are similar to those in COVID-19 patients (72). In one preprint study, hamsters infected with the SARS-CoV-2 WA1/2020, the Alpha variant, the Beta variant, and the Delta variant quickly experienced weight loss. In contrast, those infected with two different Omicron variant challenge doses maintained their weight, even at doses that were 100-times higher than the doses of other strains. In contrast to WA1/2020 infection, higher viral loads in the nose and lower viral loads in the lung were found in hamsters infected with the Omicron variant (73). These data suggest that the Omicron variant may lead to more potent upper-respiratory tract infection but less severe lower-respiratory tract infection compared with prior SARS-CoV-2 variants.

## 4 Omicron Variant: Striking Reduction in Neutralization Activity

As neutralization antibodies are thought to play an important role in protection against SARS-CoV-2 infection, concern is growing worldwide regarding the neutralization activity against the Omicron variant (74, 75). The reasonable assumption of the impact caused by the single mutation on resistance to neutralization was discussed previously. We next summarize the neutralization efficiency of polyclonal and monoclonal antibodies through either live virus-based or pseudotyped virus assays.

### 4.1 Neutralization Activity of Polyclonal Antibodies

**Figure 3:**
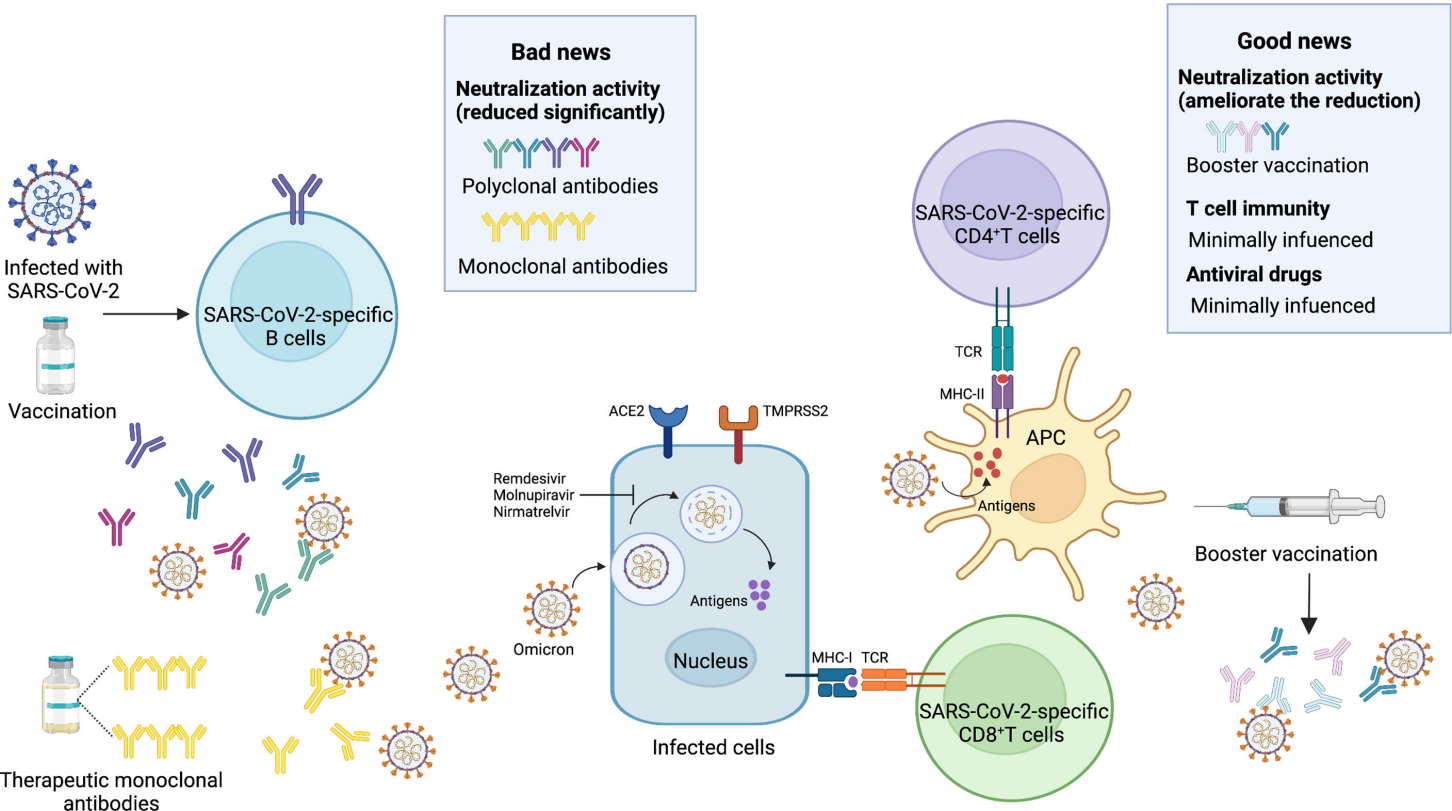
Bad news and good news about the Omicron variant. Bad news, the activity of neutralizing antibodies elicited by the SARS-CoV-2 infection and vaccines is reduced significantly. Most of the therapeutic monoclonal antibodies lose the neutralization activity. Good news, the booster vaccination can ameliorate the significant reduction. T cell immunity is minimally influenced. Antiviral drugs such as Remdesivir, Molnupiravir, Nirmatrelvir are less affected by the Omicron variant. SARS-CoV-2, severe acute respiratory syndrome coronavirus 2; ACE2, angiotensin-converting enzyme 2; TMPRSS2, transmembrane serine protease 2; MHC-I, major histocompatibility complex class I; MHC-II, major histocompatibility complex class II; TCR, T-cell receptor; APC, antigen presenting cell. Image created by Yifei Guo using BioRender (https://biorender.com/).

It has been corroborated that polyclonal antibodies, namely, the infection or vaccine-induced neutralizing antibodies, show a significant and approximately equivalent reduction in neutralization activity against the Omicron BA.1 and BA.2 variants (15, 20, 21) (**Tables 1**, **2** and **Figure 3**).

**Table 2:** Neutralization activity of polyclonal antibodies against the Omicron variant.

Convalescent plasma or sera/Vaccine name (Manufacturer)	Platform	Neutralization assay	Fold reduction/Proportion^a^(Omicron/Reference strain)	Reference strain	Characteristic of participants	Sampling time point (After Infection/2nd dose^b^)	Ref.
Convalescent plasma or sera	–	Live virus	> 11.1-fold	WT	Infected with ancestral strain in New York (N=15)	~ 58 days	(76)
	–	Live virus	~ 33.8-fold	Victoria	Infected with Alpha (N=18)	~ 42 days	(60)
	–	Live virus	~ 11.8-fold	Victoria	Infected with Beta (N=14)	~ 61 days	(60)
	–	Live virus	~ 3.1-fold	Victoria	Infected with Gamma (N=16)	~ 63 days	(60)
	–	Live virus	~ 1.7-fold	Victoria	Infected with Delta (N=42)	~ 38 days	(60)
	–	Live virus	~ 10.6-fold	WT	Infected before the pandemic of the Omicron variant	143-196 days	(77)
	–	Pseudotyped virus	> 32-fold	WT (D614G)	Presumably infected with WT (N=10)	9-120 days	(59)
	–	Pseudotyped virus	58 ± 51-fold	WT	Infected early in the pandemic (N=20)	~1.2 months	(78)
	–	Pseudotyped virus	32 ± 23-fold	WT	Infected early in the pandemic (N=20)	~ 6 months	(78)
	–	Pseudotyped virus	43 ± 23-fold	WT	Infected early in the pandemic (N=20)	~ 12 months	(78)
	–	Pseudotyped virus	~ 44-fold	Delta	Mild or severe patients (N=17)	< 2 months	(79)
BNT162b2 (Pfizer–BioNTech)	mRNA	Live virus	~ 22-fold	WT (D614G)	Infected (N = 13) Vaccinated only (N=6)	~ 26 days	(24)
		Live virus	6%/100%	WT (D614G)	Vaccinated only (N=16)	~ 5 months	(80)
		Live virus	> 23.3-fold	WT	Vaccinated only (N=10)	~ 18 days	(76)
		Live virus	~ 13.7-fold	WT	Infected with ancestral strain in New York (N=10)	~ 26 days	(76)
		Live virus	~ 14.9-fold	WT	Vaccinated only (N=20)	~ 165.6 days	(81)
		Live virus	~ 29.8-fold	Victoria	Vaccinated only (N=21)	~ 28 days	(82)
		Live virus	~ 31.3-fold	WT	Vaccinated only (N=31)	3-5 weeks	(77)
		Pseudotyped virus	~ 37-fold	WT (D614G)	Vaccinated only (N=17)	2-4 weeks	(62)
		Pseudotyped virus	~ 122-fold	WT	Vaccinated only (N=21)	< 3 months	(83)
		Pseudotyped virus	~ 12-fold	WT	Infected (N=27)	6-12 months	(83)
		Pseudotyped virus	~ 33.8-fold	WT (D614G)	Vaccinated only (N=11)	< 3 months	(79)
		Pseudotyped virus	> 21-fold	WT (D614G)	Infected (N =1) Vaccinated only (N=12)	15-213 days	(59)
mRNA-1273 (Moderna)	mRNA	Live virus	~ 42.6-fold	WT	Vaccinated only (N=10)	~ 26 days	(79)
		Live virus	~ 10.6-fold	WT	Infected with ancestral strain in New York (N=10)	~ 20 days	(76)
		Pseudotyped virus	~ 39-fold	WT (D614G)	Vaccinated only (N=14)	2-4 weeks	(62)
		Pseudotyped virus	~ 43-fold	WT	Vaccinated only (N=24)	< 3 months	(83)
		Pseudotyped virus	~ 9-fold	WT	Infected (N=8)	6-12 months	(83)
		Pseudotyped virus	> 8.6-fold	WT (D614G)	Vaccinated only (N=12)	6-177 days	(59)
AZD1222 (AstraZeneca)	Adenoviral vector	Live virus	11%/89%	WT (D614G)	Vaccinated only (N=18)	~ 5 months	(80)
		Pseudotyped virus	~ 21-fold	WT (D614G)	Vaccinated only (N=17)	2-4 weeks	(62)
		Pseudotyped virus	0%/20%	WT (D614G)	Infected (N =1) Unkown (N=4)	91-159 days	(59)
Ad26.COV2-S (Janssen Pharmaceutical)	Adenoviral vector	Live virus	~ 13.3-fold	Victoria	Vaccinated only (N=22)	~ 28 days	(82)
		Pseudotyped virus	8%/83.3%	WT (D614G)	Vaccinated only (N=12)	1-19 weeks	(62)
		Pseudotyped virus	22.2%/55.6%	WT (D614G)	Infected (N =2) Vaccinated only (N=3) Unkown (N=4)	50-186 days	(59)
		Pseudotyped virus	~ 17-fold	WT	Infected (N=6)	6-12 months	(83)
Sputnik V (Gamaleya)	Adenoviral vector	Pseudotyped virus	9%/81.8%	WT (D614G)	Vaccinated only (N=11)	2-4 weeks	(62)
BBIBP-CorV (Sinopharm)	Inactivated virus	Pseudotyped virus	57%/100%	WT (D614G)	Vaccinated only (N=13)	2-4 weeks	(62)
CoronaVac (Sinovac Biotech)	Inactivated virus	Live virus	~ 6.5-fold	WT	Vaccinated only (N=30)	3-5 weeks	(77)

WT, wild type SARS-CoV-2; ~, around.

a Proportion of detectable neutralization titers against the Omicron variant.

b All samples were collected after 2nd dose except for the 1st Ad26.COV2-S.

The Omicron variant displayed a 1.7-fold to 58-fold reduced neutralization activity to convalescent plasma or sera (59, 60, 78, 79). In one live virus-based neutralization study, samples from 40 convalescent subjects were collected at a median of 6 and 12 months post-symptom onset, and only 36% (at 6 months) and 39% (at 12 months) remained active against the Omicron variant, whereas 91% to 94% remained active against the Delta variant (80). Similarly, the Omicron variant displayed a significant reduction (>32-fold) in activity compared with the reference WT virus containing the D614G substitution (59). Furthermore, the association between variants and the neutralization activity was observed in a recent pseudotyped virus assay, which resulted in 33.8-fold, 11.8-fold, 3.1-fold, and 1.7-fold reduced activity to sera obtained from convalescent participants infected with the Alpha, Beta, Gamma, and Delta variants, respectively (60). A similar study showed that only 0 in 10 (infected with Alpha), 1 in 8 (infected with Beta), and 1 in 7 (infected with Delta) serum samples exhibited detectable neutralization titers against the Omicron variant (84). It is indicated that pre-infection with any previous VOCs can result in breakthrough infections with high risks in this wave of the Omicron variant. Notably, despite the substantial extent of evasion of an infection-induced neutralizing response against the Omicron variant, most convalescent individuals who had received a primary vaccination series (involving mRNA-1273, BNT162b2, and Ad26.COV2-S) for 6 to 12 months had detectable neutralization titers, whereas only a minimal percentage of vaccinated-only participants remained active against the Omicron variant (83). This finding was in line with another study on convalescent individuals who had received one dose of BNT162b2 or CoronaVac (77).

As vaccination is generally accepted to be one of the best ways to control the pandemic, an increasing number of studies have explored the neutralization activity of widely used vaccines. Recent studies have suggested that there is a significant impact on the neutralization activity of sera or plasma from participants receiving vaccines. In one study, sera from two doses of BNT162b2 at five months showed no neutralization activity against the Omicron variant except one sample (80). Other *in vitro* studies have shown a 12-fold to 122-fold reduced neutralization activity to the Omicron variant compared with the reference virus (24, 59, 62, 76, 77, 79, 81–83). Another mRNA-based vaccine (i.e., mRNA-1273) showed an 8.6-fold to 42.6-fold reduction in neutralization activity (59, 62, 76, 83). Other common approved vaccines, including AZD1222, Ad26.COV2-S, BBIBP-CorV, Sputnik V, and CoronaVac, also exhibited dramatic impairment in neutralizing capacity (59, 62, 77, 78, 80, 83). These data were supported initially by one clinical study, and the effectiveness of vaccination with BNT162b2 against hospitalization was 70% [95% confidence interval (CI), 62 to 76] during the proxy Omicron period but was 93% (95% CI, 90 to 94) before this period (85).

Nonetheless, the booster vaccination, which did reduce the risk of SARS-CoV-2 infection, could reverse the trend of compromised neutralization (86, 87) (**Table 3**). Of sera samples obtained from individuals receiving a third-dose mRNA vaccine, only moderately reduced neutralization activity was detected compared with the WT virus. In comparison with the non-boosted vaccinees, vaccinees boosted with mRNA-1273 and BNT162b2 had 19-fold and 27-fold increased neutralization titers, respectively (83). Booster vaccines also enhanced the neutralization activity among patients with cancer and pregnant women (88, 92). Interestingly, in contrast to the homologous and booster vaccinations, the heterologous booster vaccination appears to induce higher neutralization titers (77, 79, 90). Moreover, a longer interval between the second dose and the booster dose also contributes to higher neutralizing antibody titers against the Omicron variant (93). However, it is not clear how beneficial the fourth dose of vaccine to target the Omicron variant will be. A recent study showed that the antibody response to the fourth dose of mRNA vaccine was slightly higher than to the third dose, suggesting that maximal immunogenicity was achieved after three doses of mRNA vaccines (94).

**Table 3:** The impact of the booster vaccination on neutralization activity against the Omicron variant.

Vaccination regimen		Neutralization assay	Fold change^a^/Proportion^b^ (Booster/2nd dose^c^)	Referencetime point (After 2nd dose^d^)	Sampling time point (After Booster)	Characteristic of participants	Ref.
Primary vaccination series	Booster						
BNT162b2 (×2)	BNT162b2 (×1)	Live virus	100%/6%	~ 5 months	~ 1 month	Vaccinated only (N=20)	(80)
	BNT162b2 (×1)	Live virus	_↓_7.5-fold/_↓_23.3-fold	~ 18 days	~ 19 days	Vaccinated only (N=10)	(76)
	BNT162b2 (×1)	Live virus	_↓_13.1-fold/_↓_13.7-fold	~ 26 days	~ 20 days	Infected with ancestral strain in New York (N=10)	(76)
	① mRNA-1273 (×1) ② BNT162b2 (×1)	Pseudotyped virus	_↓_4-fold/_↓_122-fold	~ 13 days	~ 49 days	Vaccinated only (➀: N=6; ➁: N=24)	(83)
	BNT162b2 (×1)	Pseudotyped virus	_↓_8.1-fold/_↓_33.8-fold	< 3 months	< 3 months	Vaccinated only (N=10)	(79)
	BNT162b2 (×1)	Live virus	_↓_8.3-fold/_↓_14.9-fold	~ 165.6 days	~ 25 days	Vaccinated only (N=20)	(81)
	BNT162b2 (×1)	Pseudotyped virus	100%/30%	~ 1 month	~ 1 month	Vaccinated only (N=30)	(88)
mRNA-1273 (×2)	mRNA-1273 (×1)	Live virus	_↓_16.7-fold/_↓_42.6-fold	~ 26 days	~ 19 days	Vaccinated only (N=10)	(76)
	① mRNA-1273 (×1) ② BNT162b2 (×1)	Pseudotyped virus	_↓_6-fold/_↓_43-fold	~ 18 days	~ 21 days	Vaccinated only (➀: N=32; ➁: N=1)	(83)
	mRNA-1273 (×1)	Pseudotyped virus	_↓_6.5-fold/_↓_35.1-fold	~ 14 days	~ 14 days	Infected (N =2) Vaccinated only (N=5)	(89)
BBIBP-CorV (×2)	BBIBP-CorV (×1)	Pseudotyped virus	_↓_5.9-fold/_↓_11.2-fold	~ 14 days	~ 14 days	Vaccinated only (N=10)	(90)
	ZF2001 (×1)	Pseudotyped virus	_↓_14.98-fold/_↓_11.2-fold	~ 14 days	~ 14 days	Vaccinated only (N=10)	(90)
	BBIBP-CorV (×1)	Pseudotyped virus	78.08%/25.68%	~ 28 days	~ 28 days	Vaccinated only (N=292)	(91)
CoronaVac (×2)	CoronaVac (×1)	Live virus	63.3%/0%	3-5 weeks	3-5 weeks	Vaccinated only (N=30)	(77)
	BNT162b2 (×1)	Live virus	100%/0%	3-5 weeks	3-5 weeks	Vaccinated only (N=30)	(77)
mRNA-1273 (×2) BNT162b2 (×2)	① mRNA-1273 (×1) ② BNT162b2 (×1)	Pseudotyped virus	_↓_5.1-fold/_↓_21.3-fold	31-121 days	2-112 days	Patients with cancer (➀: N=9; ➁: N=18)	(88)

↓, reduction; ~, around; ×2, 2 doses; ×1, 1 dose.

a Fold change in neutralization activity against the Omicron variant compared with the wild-type SARS-CoV-2.

b Proportion of detectable neutralization titers against the Omicron variant.

c,d All samples were collected after 2nd dose except for the 1st Ad26.COV2-S.

Collectively, the Omicron variant has a great impact on the humoral immunity elicited by infection and vaccines, although the booster vaccination can ameliorate this reduction to some extent.

### 4.2 Neutralization Activity of Monoclonal Antibodies in Clinical Use

Several mAbs, most of which target the RBD, have been approved for clinical use. The anti-RBD mAbs are classified into four classes (class I, class II, class III, and class IV) according to the RBD binding characteristics (95). Combination with different classes of mAbs, also known as the cocktail of antibodies, is considered to be a strategy to suppress the immune escape effect. For the Omicron BA.1 variant, there is evidence that most therapeutic mAbs partly and even completely lose the neutralization activity. Class I and class II mAbs, involving CB6 (etesevimab), REGN10933 (casirivimab), COV2-2196 (tixagevimab), CT-P59 (regdanvimab), Brii196 (lamubarvimab), and LY-CoV555 (bamlanivimab) exhibited more than 100-fold reduced potency or completely lost the neutralization activity, as did some class III antibodies, including REGN10987 (imdevimab) and COV2-2130 (cilgavimab). Likewise, antibodies in combination, such as the REGN-COV2 cocktail (REGN10933+ REGN10987), Lily cocktail (LY-CoV555+CB6), and AstraZeneca’s antibody cocktail (COV2-2130+COV2-2196) experienced significantly reduced neutralization activity (**Figure 3**). Conversely, the Omicron variant escapes from most mAbs, but S309 (sotrovimab), which is a class III mAb, still retains potent neutralization activity because of its ability to target one highly conserved epitope on the RBD (35, 59, 60, 62, 79, 80, 96, 97). Considering the Omicron BA.2 variant has 4 unique mutations in the RBD, it is reasonable to conclude that there will be differences in the effectiveness of therapeutic mAbs from the Omicron BA.1 variant. Recent laboratory neutralization assays have shown that sotrovimab is largely inactive whereas cilgavimab and AstraZeneca’s antibody cocktail retain neutralization activity (15–18) (**Table 1**). Taken together, none of the current therapeutic monoclonal antibodies retain neutralization activity to all variants, although sotrovimab possesses the ability to defend against numerous mutations (92). In light of adaptive variants, it may be a more effective strategy to screen mAbs binding to relatively conserved epitopes.

## 5 Omicron Variant: Good News Exists Beyond “neutralization”

The neutralization of SARS-CoV-2, which is dependent on the Fab fragments of antibodies, can protect against infection, but it does not, on its own, solve the problem of viral clearance. Another defense mechanism of antibodies is the activation of immune cells bearing Fc receptors. It has been reported that Fc-effector functions contribute to reducing the burden of SARS-CoV-2 and alleviating lung inflammation in hamster and mice models (98, 99). Moreover, mRNA vaccine-induced antibodies retain robust Fc-effector functions against VOCs, which may partially account for less severe symptoms among vaccinees (100, 101). Further studies are required to elucidate the impact of Fc-mediated responses elicited by infection and vaccination against the Omicron variant.

In contrast to the neutralization antibodies, the non-neutralizing antibodies against the influenza virus also have demonstrated that they play a key role in protection according to their Fc-mediated responses (102, 103). Indeed, a large number of non-neutralizing antibodies can broadly cross-react with coronaviruses (104). Whether or not the non-neutralizing antibodies of the Omicron variant infection exhibit similar functions in influenza infection, however, remains unclear.

In addition to B-cell immunity, which is mainly oriented toward secretion of high-affinity neutralizing antibodies, the function of T-cell immunity in SARS-CoV-2 infection cannot be neglected. Prior studies have demonstrated that both infection-induced and vaccine-induced T-cell immunity are less affected by mutations and retain their ability to prevent severe COVID-19 cases (105–108). Whether or not the preexisting and cross-reactive T-cell immunity still work is of significant concern. Through an initial analysis of the epitopes in the Omicron variant, 94.4% of CD8^+^T cell epitopes and 90% of CD4^+^T cell epitopes were fully conserved, which indicated that T-cell immunity was spared (109). In another study, T-cell response to the Omicron variant was detected in individuals with a prior history of SARS-CoV-2 infection or BNT162b2 vaccination. In addition, 84% spike-specific CD4^+^T cells and 70% spike-specific CD8^+^T cells were detectable in convalescent patients. Notably, 91% spike-specific CD4^+^T cells and 92% spike-specific CD8+T cells were detectable in vaccinees. In the same study, the function and phenotype of T cells (involving CD4^+^T cells and CD8^+^T cells) were examined, and in accordance with the frequencies, they were not affected (110) (**Figure 3**). Besides, booster vaccines may facilitate a more robust T-cell immunity (111).

Because antiviral drugs target RNA-dependent RNA polymerase (RdRp) and the main protease bearing fewer mutations in the Omicron variant, the accumulated evidence has demonstrated that the antiviral activity of remdesivir, molnupiravir, and nirmatrelvir against the Omicron variant including BA.1 and BA.2 is similar to its activity against other variants (16, 96, 112) (**Table 1** and **Figure 3**).

## 6 Conclusion

Battling the Omicron variant involves guesswork. Vaccination is the best way to prevent COVID-19, particularly severe diseases, although the neutralization activity against the Omicron variant elicited by current vaccines has been a substantial reduction. Moreover, among individuals who had recovered from infection with the Omicron variant without previous SARS-CoV-2 infection, serum from vaccinated persons exhibited high levels of neutralization titers against all the VOCs whereas serum from unvaccinated persons mainly showed neutralization activity against the Omicron variant (113, 114).Therefore, with the emergence of recombination lineages - XD, XF and XE, it should not be neglected the role of vaccination in cross-reactive neutralization (13). In addition, given the SARS-CoV-2 infection belongs to the mucosal infection and higher viral loads of the Omicron variant in the nose than other variants, novel mucosal vaccines possess the potential to elicit effectively protective immune responses in the first place (115).

## Author Contributions

All authors contributed equally to the concept and preparation of the manuscript. YFG, JHan, and JZ completed the final preparation and editing of the manuscript. YFG created the figures and tables. All authors contributed to the article and approved the submitted version.

## Funding

This work was supported by the National Natural Science Foundation of China under Grant No. 81871640, 82172255, Shanghai Shen Kang Hospital Development Center under Grant No. SHDC12019116 and Shanghai Key Clinical Specialty Construction Program under Grant No. ZK2019B24.

## Conflict of Interest

The authors declare that the research was conducted in the absence of any commercial or financial relationships that could be construed as a potential conflict of interest.

## Publisher’s Note

All claims expressed in this article are solely those of the authors and do not necessarily represent those of their affiliated organizations, or those of the publisher, the editors and the reviewers. Any product that may be evaluated in this article, or claim that may be made by its manufacturer, is not guaranteed or endorsed by the publisher.
